# Supplementary material for: Collaborative intelligence in AI: Evaluating the performance of a council of AIs on the USMLE
Source: PLOS Digit Health. 2025 Oct 9;4(10):e0000787. doi: 10.1371/journal.pdig.0000787 (PMC12510544; doi:10.1371/journal.pdig.0000787)
Supplement: S2 File — (ZIP) [file pdig.0000787.s002.zip › S2 Data Raw data files containing unprocessed question inputs and LLM outputs including transcripts of deliberations/STEP3_Question-123-1_Type-MC-NJ.docx]

**Facilitator** **[Round 1]:** A 55-year-old man is brought to the emergency department by his wife because he told her he did not want to live anymore. During the past 6 weeks, he has experienced fatigue, loss of interest in usual activities, a 7-kg (15-lb) weight loss, and insomnia. He has no history of serious medical or psychiatric illness. Vital signs are temperature 37.0°C (98.6°F), pulse 80/min, respirations 16/min, and blood pressure 140/82 mm Hg. Physical examination discloses no abnormalities. Beck Depression Inventory score is 35 (severely depressed). He says he feels guilty that his investments have not done well and that he has ruined his family finances. He reports he hears voices at night telling him he has sinned. In addition to olanzapine, which of the following is the best treatment option for this patient? (A) Divalproex (B) Fluoxetine (C) Lamotrigine (D) Lithium carbonate (E) No other therapy is necessary

**AI_D:** RESTATING THE QUESTION: Here we have a patient, a 55-year-old man who is reportedly facing severe symptoms of depression evidenced by his Beck Depression Inventory score of 35. Along with his depressive symptoms, he is also experiencing auditory hallucination as he hears voices at night. The question is asking for the best additional treatment option for this patient once olanzapine has been administered. PLANNING: Olanzapine is an atypical antipsychotic used in the management of symptoms such as hallucinations, delusions and paranoia often seen in disorders such as schizophrenia. However, this man also displays severe symptoms of depression. The medications listed are primarily anticonvulsants (Divalproex, Lamotrigine) and mood stabilizers (Lithium carbonate) with the exception of Fluoxetine, a selective serotonin reuptake inhibitor (SSRI) which is primarily used as an antidepressant. CLINICAL REASONING: Depression is an underlying theme in this patient's presentation. He is suffering from a severe loss of interest, weight loss, insomnia, guilt, and has even expressed suicidal ideations. Fluoxetine, by blocking the reuptake of serotonin into presynaptic cells, can improve the symptoms of depression. CONCLUDING: This patient is on olanzapine for his hallucinatory experiences, however, his severe depression cannot be managed effectively by olanzapine alone. Adding on an antidepressant such as Fluoxetine can effectively tackle his depressive symptoms and thus improve his overall mental wellness. RESPONSE: Therefore, the best treatment option in addition to Olanzapine for this patient, is Fluoxetine (B).

**AI_C:** RESTATING THE QUESTION: The query is about a 55-year-old man, with no past critical medical or mental health ailments, who's been experiencing severe symptoms of depression, specifically fatigue, disinterest in usual activities, substantial weight loss, insomnia, guilt related to financial situation, and auditory hallucinations. The clinical approach requires us to understand which therapy would best supplement olanzapine in his treatment. PLANNING: In this context, the alternate assumptions can include considering whether the patient's symptoms require antipsychotic, antidepressant, mood stabilizing or no additional therapy. Factors such as hallucinations, high Beck's Depression Inventory score, and various depressive symptoms suggest significant severity prompting the need for adjunct therapy in addition to olanzapine. CLINICAL REASONING: The patient's severe depression is underscored-by his high score on the Beck Depression Inventory and his symptoms of significant weight loss, insomnia, anhedonia, and fatigue. Moreover, he's hearing voices can suggest psychotic features. These symptoms necessitate the use of adjunctive therapy with an antipsychotic such as Olanzapine. Among the options given, the effectiveness of antidepressants in managing severe depression is substantial, particularly when psychotic features are also present. CONCLUDING: Fluoxetine, of the group of selective serotonin reuptake inhibitors (SSRIs), has a high efficacy in treating major depressive disorder with psychotic features. Its pharmacological benefit lies in balancing serotonin levels, thereby improving depression symptoms and patient's mood. RESPONSE: Given the severity of the patient's depressive symptoms along with hallucinations, the best adjunctive therapeutic option to treat this patient in addition to Olanzapine would be the antidepressant Fluoxetine (Option B).

**AI_E:** RESTATING THE QUESTION: The query involves the management of psychiatric illness in a 55-year-old individual, in addition to olanzapine. This person is experiencing severe depression, as indicated by a high Beck Depression Inventory score, and has suicidal ideation. Symptoms include significant weight loss, fatigue, anhedonia, insomnia, guilt regarding faulty investments, and auditory hallucinations. The ultimate question revolves around the best treatment option available for this individual among the options: Divalproex, Fluoxetine, Lamotrigine, Lithium carbonate, or no further treatment. PLANNING: In order to determine which treatment option should be clinically prioritized, we need to focus on the patient's primary symptoms. The patient's report of hearing voices indicating he has sinned requires further consideration. This symptom, in combination with severe depressive symptoms, suggests the presence of a "psychotic depression." We would conventionally treat psychotic depression with a combination of an antipsychotic and an antidepressant. Therefore, treatment selection should focus on the options that include antidepressants. CLINICAL REASONING: In the context of a patient with psychotic depression, our focus should be on combining an antipsychotic like olanzapine with an antidepressant. Neither Divalproex (a mood stabilizer used for bipolar disorder), Lamotrigine (a mood stabilizer and antiepileptic), nor Lithium carbonate (a mood stabilizer predominantly used for bipolar disorder, particularly manic episodes) meet this requirement. Fluoxetine, on the other hand, is a selective serotonin reuptake inhibitor (SSRI) which is commonly used for the treatment of major depressive disorder. The option of no further treatment is inappropriate given the severity of the patient's symptoms and the risk of suicide. CONCLUDING: Considering the patient's severe depressive symptoms coupled with hallucinatory experiences, a treatment plan combining an antipsychotic (olanzapine) and an antidepressant is most relevant. Among the options provided, Fluoxetine aligns the most in this case. Therefore, we can conclude that fluoxetine would be an excellent additional therapy in this patient's treatment regimen. RESPONSE: The best treatment choice for this patient, in addition to olanzapine, would be Fluoxetine (Option B).

**AI_B:** RESTATING THE QUESTION: The scenario outlines a 55-year-old male showing severe depressive symptoms including suicidal ideation, guilt about financial mismanagement, lamenting about a physiological state of fatigue, weight loss, insomnia, and auditory hallucinations, which are all symptoms characteristic of major depressive disorder (MDD) with psychotic features. The query asks which medication is recommended in addition to olanzapine, an atypical antipsychotic, to manage this person's condition. PLANNING: The assumptions here are that this person suffers from MDD with psychotic features, and that olanzapine alone may not be sufficient to manage his symptoms. The alternate assumption would be that combining olanzapine with another antidepressant medication could provide a more comprehensive treatment approach addressing both the depressive and psychotic symptoms. To clinically prioritize, one should favor the medication known to exhibit better efficacy and fewer side effects in treating the said condition. CLINICAL REASONING: Management of MDD with psychotic features often involves the use of both an antipsychotic AND an antidepressant medication. This is due to the fact that antipsychotics primarily target psychotic symptoms (in this case, the auditory hallucinations or "voices"), while antidepressants aim to alleviate depressive symptoms (such as loss of interest, guilt feelings, insomnia, etc.). Hence, evidence suggests that combination therapy (antipsychotic+antidepressant) is superior to antipsychotic monotherapy in the treatment of this condition. CONCLUDING: Of the treatment options provided, fluoxetine (a selective serotonin reuptake inhibitor or SSRI) is the only one that fits into the class of antidepressants. Divalproex, lamotrigine, and lithium carbonate are more commonly used in managing bipolar disorder. Hence, the most appropriate additional medication in our patient's case would be an SSRI like fluoxetine, aiming to manage his depressive symptoms. RESPONSE: Therefore, the best additional treatment option for this patient, given the indications and his condition, is (B) fluoxetine.

**AI_A:** 1) RESTATING THE QUESTION: The inquiry presents a situation concerning a 55-year-old man presenting with potential symptoms of severe depression, including fatigue, lack of interest, significant weight loss, insomnia, a feeling of guilt over his financial situation, and auditory hallucinations at night, which could be indicative of a psychotic feature. After treatment with olanzapine, an antipsychotic agent, we're asked to identify the most effective additional treatment from the following options: Divalproex, Fluoxetine, Lamotrigine, Lithium carbonate, or no further treatment. 2) PLANNING: Few underlying assumptions to address include: whether depression is truly the patient's main diagnosis; whether the psychotic feature requires additional treatment beyond olanzapine; the efficacy and appropriateness of each drug presented in further managing his symptoms. From these, the most clinically relevant would seem to be the question of which medication would best address his severe depression with potential psychotic features. 3) CLINICAL REASONING: In processing this case of a severely depressed, potentially psychotic patient, we must assess the treatments presented for their relevance to his symptoms and safety given his physical health. His symptoms point towards Major Depressive Disorder with Psychotic Features, which typically requires the combination of an antipsychotic and an antidepressant. Olanzapine is applied to alleviate his psychotic symptoms. Now, we need an antidepressant to address the depressive symptoms. Among the options, Fluoxetine, a selective serotonin reuptake inhibitor (SSRI), is a common and effective antidepressant for treating depression. The other medications - Divalproex, Lamotrigine, and Lithium carbonate - are more associated with mood stabilization in bipolar disorders, while our patient does not have any such report. 4) CONCLUDING: After a step-by-step evaluation of clinical findings and therapeutic intervention options, it is evident that the patient's symptoms are aligned with Major Depressive Disorder with Psychotic Features. Recognizing the patient has already been on an antipsychotic, i.e., olanzapine, the clinical thought process leads us to explore the option of adding an antidepressant to this regimen. Considering the options at hand, fluoxetine - an SSRI, is most appropriate. 5) RESPONSE: Thus, for this 55-year-old man reporting major depressive symptoms along with auditory hallucinations, the best treatment option beyond the already prescribed Olanzapine would be (B) Fluoxetine. This choice would ensure a holistic approach catering to both the depressive and psychotic aspects of his condition.

**Facilitator** The consensus statement synthesizes the responses of AI_A, AI_B, AI_C, AI_D, and AI_E as follows: The question presents a 55-year-old man with severe symptoms of depression, including fatigue, loss of interest, significant weight loss, insomnia, guilt over financial mismanagement, and auditory hallucinations, indicative of Major Depressive Disorder with Psychotic Features. The inquiry asks for the most effective additional treatment to olanzapine, an antipsychotic agent, from the options: Divalproex, Fluoxetine, Lamotrigine, Lithium carbonate, or no further treatment. The underlying assumptions include the patient's primary diagnosis of depression with potential psychotic features and the need for additional treatment beyond olanzapine. The clinical reasoning process involves assessing the relevance and safety of the treatments presented for his symptoms. The patient's symptoms necessitate the use of an antipsychotic like olanzapine and an antidepressant. Among the options, Fluoxetine, a selective serotonin reuptake inhibitor (SSRI), is a common and effective antidepressant for treating depression. The other medications - Divalproex, Lamotrigine, and Lithium carbonate - are more associated with mood stabilization in bipolar disorders, which the patient does not report. After a step-by-step evaluation of clinical findings and therapeutic intervention options, it is evident that the patient's symptoms align with Major Depressive Disorder with Psychotic Features. Recognizing the patient has already been on an antipsychotic, i.e., olanzapine, the clinical thought process leads us to explore the option of adding an antidepressant to this regimen. Considering the options at hand, fluoxetine - an SSRI, is most appropriate. Therefore, for this 55-year-old man reporting major depressive symptoms along with auditory hallucinations, the best treatment option beyond the already prescribed Olanzapine would be (B) Fluoxetine. This choice would ensure a holistic approach catering to both the depressive and psychotic aspects of his condition.
